# Patient-reported outcomes in randomized controlled trials of spinal disorders: a methodological quality assessment and recommendations for future research

**DOI:** 10.1530/EOR-2025-0171

**Published:** 2026-06-01

**Authors:** Wenbo He, Rui Liu, Jinyi Ma, Weihan Hou, Zhengchi Li, Liang Du

**Affiliations:** ^1^ Innovation Institute for Integration of Medicine and Engineering, Chinese Evidence-Based Medicine Center, West China Hospital, Sichuan University, Chengdu, China; ^2^ Rehabilitation Medicine Center and Institute of Rehabilitation Medicine, West China Hospital, Sichuan University, Chengdu, Sichuan, PR China; ^3^ Key Laboratory of Rehabilitation Medicine in Sichuan Province, Chengdu, Sichuan, PR China; ^4^ Center for Education of Medical Humanities, West China Hospital, Sichuan University, Chengdu, China; ^5^ West China Medical Center, Sichuan Medical University, Chengdu, China

**Keywords:** patient-reported outcomes, randomized controlled trials, spinal disorders, reporting quality, methodological quality assessment

## Abstract

**Purpose:**

- This study aimed to assess the types of PRO instruments used and evaluate the overall quality of PRO reporting in RCTs addressing spinal disorders.

**Methods:**

- A systematic literature search of PubMed and Web of Science was conducted to identify RCTs reporting PROs in spinal conditions. Trials were screened according to PRISMA guidelines, and the quality of PRO reporting was evaluated using the ISOQOL-recommended checklist. Descriptive analyses and multivariable linear regression were performed to explore predictors of reporting quality.

**Results:**

- A total of 42 RCTs involving 8,669 participants were included. Most trials (95.2%) designated PROs as primary outcomes, with lumbar degenerative diseases being the most common focus (45.2%). Across studies, 25 distinct PRO tools were identified and grouped into five domains: pain, functional disability, quality of life, patient experience, and psychological assessment. The Visual Analog Scale (VAS) and Oswestry Disability Index (ODI) were the most frequently used instruments. However, tools assessing psychological domains were underutilized. Reporting adherence was higher when PROs were primary outcomes, but only 27.2% of studies met high-quality reporting standards. Critical deficiencies were noted in justifying instrument selection, data collection modalities, and handling of missing data. Regression analysis revealed no statistically significant predictors of reporting quality.

**Conclusion:**

- Despite the widespread inclusion of PROs in spinal disorder RCTs, major methodological gaps persist in reporting practices. Enhancing standardization in PRO tool selection, data collection methods, and statistical handling of missing data is essential for improving the validity, comparability, and clinical impact of PRO-based evidence in spinal health research.

## Introduction

Spinal disorders, including spinal cord injuries, represent a major global health burden and are among the leading contributors to disability-adjusted life years (DALYs) worldwide, with spinal cord injuries also associated with increased mortality risk (1, 2). Beyond physical limitations, patients frequently experience chronic pain, neurological deficits, and significant psychosocial stress, which can lead to anxiety, depression, and impaired social participation (3, 4). These multidimensional impairments not only reduce treatment adherence but also profoundly diminish patients’ long-term quality of life (QoL), particularly by constraining their ability to work and engage socially.

Traditional outcome indicators in spinal surgery – such as surgical timing, radiographic findings, and clinician-assessed scoring systems – primarily reflect objective technical or anatomical outcomes, yet often fail to capture patients’ subjective experiences of recovery. In contrast, patient-reported outcomes (PROs), collected through validated tools such as the Oswestry Disability Index (ODI), Visual Analog Scale (VAS), and the Short-Form Health Survey (SF-36), provide direct insight into patients’ perceived symptom relief, functional recovery, and overall well-being. These instruments have become a cornerstone of value-based healthcare by aligning outcome evaluation with domains most relevant to patients (5, 6, 7). Since 2009, institutions such as the National Institute for Health and Care Excellence (NICE) and the FDA have advocated for the routine use of patient-reported outcome measures (PROMs), especially in fields such as spinal surgery, where subjective improvement is a key marker of treatment success (8).

Despite increasing incorporation of PROs into randomized controlled trials (RCTs) for spinal disorders, considerable methodological variability and reporting deficiencies persist. Common issues include inconsistent rationale for tool selection, insufficient descriptions of data collection procedures, and inadequate handling of missing data. These deficiencies undermine the comparability and reproducibility of findings, ultimately limiting their applicability to real-world clinical decision-making. Particularly in spinal care – where patients perceived functional improvement often guides treatment satisfaction – these reporting gaps hinder the broader adoption of evidence-based, patient-centered care.

Although international guidelines such as CONSORT-PRO (9) and recommendations by the International Society for Quality-of-Life Research (ISOQOL) (10) have been developed to improve the standardization and transparency of PRO reporting, their uptake in the spinal research field remains limited. Although several systematic evaluations have examined PRO reporting in fields such as oncology and general surgery (11, 12), these findings cannot be directly extrapolated to spinal research due to discipline-specific characteristics – such as the predominance of subjective functional outcomes, higher variability in disability measures, and the central role of pain-related PROs in treatment evaluation. To date, no study has systematically assessed PRO reporting quality specifically within spinal RCTs.

To address these issues, this study systematically reviews PRO reporting practices in randomized controlled trials of spinal disorders. Specifically, our objectives are to i) assess the completeness and transparency of PRO reporting using ISOQOL-recommended criteria; ii) examine the rationale, consistency, and methodological rigor underlying PRO tool selection; and iii) identify study characteristics associated with high-quality reporting. By elucidating current limitations and highlighting areas for methodological improvement, this study seeks to inform future spinal research by supporting more transparent, methodologically rigorous, and patient-centered trial practices.

## Methods

### Study design and setting

This systematic review was registered with PROSPERO (registration number: CRD420251013686) and the PRISMA guidelines for systematic reviews (13). The methodology followed the approach outlined by Claassens *et al.* (14) and adhered to the recommendations in the *Cochrane Handbook for Systematic Reviews of Interventions* (15).

### Study selection

A comprehensive literature search was conducted in PubMed, Web of Science, and ClinicalTrials.gov for peer-reviewed studies published between January 1, 2000, and January 1, 2025. The search strategy included a combination of Medical Subject Headings (MeSH) and free-text keywords related to i) patient-reported outcomes (e.g. quality of life*, *functional status, pain, depression, fatigue, emotional, psychosocial); ii) spinal conditions (e.g. spine, spinal cord injuries, central cord syndrome); iii) interventions (e.g. surgical procedures, general surgery); and iv) study design (randomized controlled trial (RCT)). The full search strategy is available in Supplementary File 1 (see section on Supplementary materials given at the end of the article). Reference lists of relevant articles were also manually screened.

Studies were included if they met the following criteria: i) RCTs involving adults (≥18 years) with spinal or spinal cord disorders; ii) comparisons involving surgical and/or nonsurgical interventions (e.g. physical therapy, pharmacologic treatments, rehabilitation); iii) use of PROs as primary or secondary endpoints; iv) evaluation of intervention impact on symptoms, functional status, health status, or quality of life; and v) sample size ≥50 participants. Exclusion criteria were i) non-English publications; ii) reviews, protocols, or duplicate records; iii) studies comparing only surgical techniques, implants, or materials without assessing PROs; iv) use of proxy-reported outcomes rather than direct patient input; and v) trials unrelated to spinal disorders or surgical decision-making.

Study selection was conducted using Covidence software (16). Two reviewers independently screened titles, abstracts, and full texts according to predefined inclusion and exclusion criteria. Discrepancies were resolved through discussion and, when necessary, consultation with a third reviewer.

### Data extraction

Data were independently extracted by two reviewers in duplicate using a standardized and pilot-tested Excel form. Discrepancies were resolved by consensus or adjudication by a third reviewer. The form included items on general trial characteristics, such as sample size, country or setting, treatment arms, PRO instruments used, and timing or methods of assessment. It also included items assessing adherence to ISOQOL recommendations for PRO reporting.

### Reporting quality assessment

A preliminary review systematically identified several tools for evaluating PRO reporting quality in RCTs, including CONSORT-PRO and SPIRIT-PRO extensions (17, 18, 19). Among them, the ISOQOL Consensus-based Recommendations for PRO Reporting of RCT Publications was selected based on their broad endorsement, methodological comprehensiveness, and relevance to PRO design and reporting (20). It offers the most comprehensive framework for evaluating trial-level PRO design and reporting, including pre-specified objectives and handling of missing data (9).

Aligned with the aims of this study, the rationale, consistency, and methodological rigor underpinning PRO tool selection were evaluated using the corresponding items embedded within the ISOQOL reporting checklist. The ISOQOL framework includes explicit criteria to determine whether authors justified their choice of PRO instruments, reported their conceptual relevance, and described psychometric properties or the appropriateness of the instruments for the targeted outcome domains (10).

Accordingly, PRO reporting quality in this study was assessed using the ISOQOL checklist, which consists of 29 items for trials where PROs were the primary endpoint and 18 items for trials where PROs were secondary or exploratory endpoints (see Supplementary File 2). Each item was independently assessed by two reviewers and scored as 1 if reported and 0 if not. Partial reporting was considered non-adherent (21). The raw scores were summed and normalized by dividing by the total number of applicable items, yielding an adjusted quality score ranging from 0 (lowest) to 100 (highest) (22, 23).

Studies were deemed high-quality if they met ≥20/29 criteria (primary endpoint) or ≥12/18 criteria (secondary endpoint), consistent with prior literature suggesting that adherence to at least two-thirds of ISOQOL recommendations indicates methodological rigor. This threshold has been widely adopted in prior PRO literature to indicate acceptable reporting quality (24, 25).

### Statistical analysis

Descriptive statistics, including medians and interquartile ranges (IQRs), were used to summarize adjusted ISOQOL checklist scores. Data distribution was assessed prior to regression analysis. Multivariable linear regression was conducted to identify factors associated with higher ISOQOL checklist scores. Covariates included i) year of publication (continuous), ii) impact factor (continued), iii) multinational study (yes/no), iv) corporate/industry funding (yes/no), v) sample size (>200 vs ≤200), vi) PRO as a primary endpoint (yes/no), vii) difference between treatment arms in the primary endpoint (yes/no), viii) follow-up PRO reporting in secondary publications (yes/no), and ix) disease type (lumbar degenerative diseases, cervical spine disorders, chronic low back pain, vertebral fractures, other).

In addition, exploratory analysis was conducted to assess whether any statistically significant differences (*P* < 0.05) in primary PRO outcomes were reported at any time point in each RCT. Model assumptions were assessed prior to regression analysis. All statistical analyses were performed using SPSS software (version 25; SPSS Inc., USA).

## Results

### Key characteristics of identified trials

**Figure 1:**
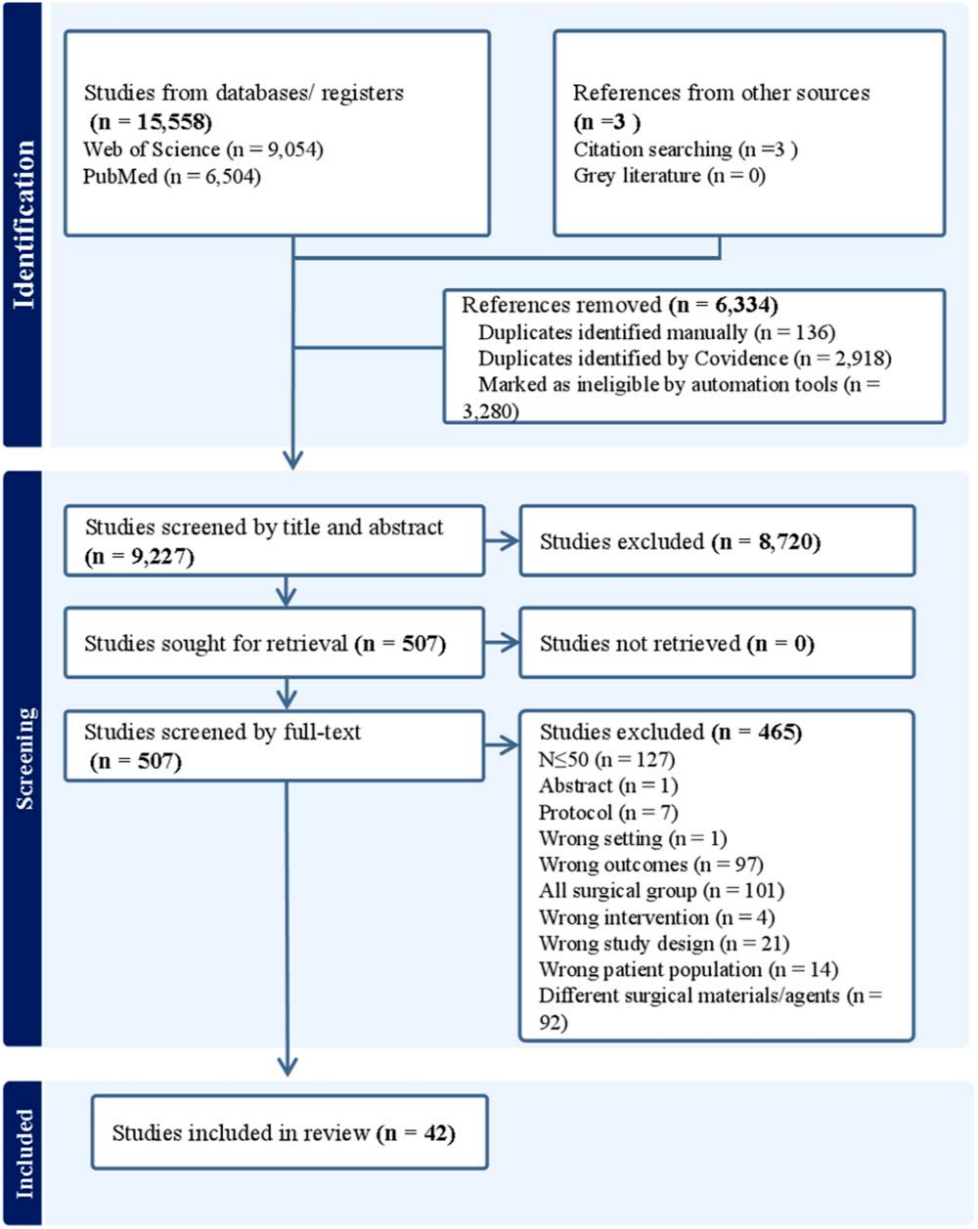
Flowchart for the inclusion and exclusion of RCTs.

A total of 15,561 records were identified (15,558 through database searches and 3 from other sources) and screened according to the PRISMA 2020 guidelines. After removal of duplicates and exclusion based on titles and abstracts, 507 full-text articles were assessed for eligibility, of which 42 met the inclusion criteria and were included in the final analysis. A PRISMA 2020-compliant flowchart detailing the study selection process is provided in Fig. 1, and a full list of included articles is provided in Supplementary File 3.

The 42 included trials involved a total of 8,669 participants, with sample sizes ranging from 48 to 474. Only four of the included RCTs (9.5%) were conducted as multicenter studies. Geographically, the majority were conducted in the United States (*n* = 19, 45.2%), followed by Norway (*n* = 4, 9.5%), Sweden (*n* = 3, 7.1%), and the United Kingdom (*n* = 3, 7.1%). The majority of RCTs (*n* = 40, 95.2%) explicitly designated PROs as primary outcomes. In addition, 19 studies (45.2%) focused on patients with lumbar degenerative diseases. The detailed characteristics of each RCT are provided in Supplementary File 4.

Statistical differences in PROs between treatment groups were observed in 38 studies (90.47%). Among these 30 studies: 8 studies (21%) reported significant differences in symptom-related outcomes only (e.g. pain, fatigue); 5 studies (13.1%) reported significant differences in non-symptom PRO domains (e.g. functional status, psychosocial outcomes); and 25 studies (65.7%) reported differences in both symptom and non-symptom PRO domains. Further details are presented in Table 1.

**Table 1:** Demographic characteristics of the randomized clinical trials.

Variable	Values, *n* (%)
Number of RCTs	42 (100.00%)
Basic RCT demographics	
Multinational study	
Yes	4 (9.52%)
No	38 (90.48%)
Overall study sample size^†^	
≤200 patients	24 (57.14%)
>200 patients	18 (42.86%)
Type of disease	
Lumbar degenerative diseases	19 (45.24%)
Vertebral fractures	10 (23.81%)
Chronic low back pain	7 (16.67%)
Cervical spine disorders	3 (7.14%)
Other spine disorders	3 (7.14%)
Region of trial published	
North America	19 (45.24%)
Europe	17 (40.48%)
Asia	5 (11.90%)
South America	1 (2.38%)
Type of intervention and control group	
Surgical vs conservative/nonsurgical	18 (42.86%)
Novel devices/interventions vs conventional conservative/drugs	15 (35.71%)
Surgery vs rehabilitation/multidisciplinary treatment	9 (21.43%)
Journal impact factor* (min, max)	1.6, 98.4
Corporate/industry supported (fully or in part)	
Yes	14 (33.33%)
No	28 (66.67%)
Difference between treatment arms in the primary endpoint	
Yes	30 (71.43%)
No	12 (28.57%)
PRO-related basic characteristics	
PRO as primary endpoint	
Yes	40 (95.24%)
No	2 (4.76%)
PRO instrument used	
ODI	32 (76.19%)
VAS for pain	26 (61.90%)
SF-36	22 (52.38%)
Satisfaction with treatment (Likert scale)	16 (38.10%)
EQ-5D	10 (23.81%)
Patient self-reported improvement (Likert scale)	10 (23.81%)
Others	59
PRO collection methods	
Electronic	1 (2.38%)
Paper	0 (0.00%)
Electronic + paper	1 (2.38%)
Not reported	40 (95.24%)
PRO difference between treatment arms	
Significant	38 (90.47%)
Nonsignificant	4 (9.53%)
Domain with statistically significant PRO difference	
Symptom-only (disease-specific scales such as pain)	8 (19.05%)
Areas other than symptoms^‡^	5 (11.90%)
Multiple domains	25 (59.52%)
Length of PRO assessment during RCT	
≤1 year	16 (38.10%)
>1 year to ≤3 years	14 (33.33%)
>3 years	12 (28.57%)
Reported PROs in a subsequent publication	
Yes	10 (23.81%)
No	32 (76.19%)

* There is no impact factor for 1 trial, and the remaining impact factors are 5-year impact factors obtained from the respective impurities home pages.

^†^ Regardless of patients included in the PRO analysis.

^‡^ Examples include physical or emotional functioning or quality of life.

### Type of PRO instruments

**Figure 2:**
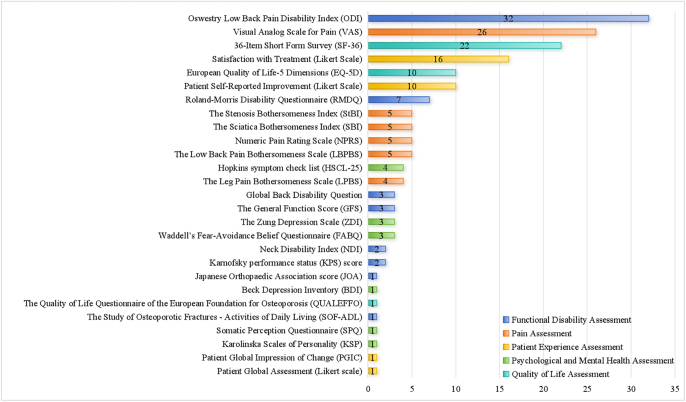
Categorization and frequency of included PRO measurement tools.

Across the 42 included RCTs, 25 distinct PRO instruments were identified and classified into five domains: i) pain assessment, ii) functional disability assessment, iii) quality-of-life assessment, iv) patient experience assessment, and v) psychological and mental health assessment (Fig. 2). Instruments targeting pain were the most frequently used, especially the Visual Analog Scale (VAS, 62%) and the numeric pain rating scale (NPRS). Region-specific scales such as the Low Back Pain Bothersomeness Scale (LBPBS) and Sciatica Bothersomeness Index (SBI) were applied in select trials, likely reflecting condition-specific needs.

#### Pain assessment

Pain was assessed using six distinct instruments, with the VAS (used in 36 studies, 86%) being the most widely adopted tool, valued for its simplicity and reliability in capturing subjective pain intensity. The NPRS was employed in 5 studies and is similarly recognized for strong psychometric performance across acute and chronic pain settings (26). Region-specific scales (e.g. LBPBS, SBI, StBI), though used in only 4–5 studies each, offer a more nuanced evaluation of pain localized to specific anatomical regions. However, the inconsistent use of these instruments suggests limited standardization across trials.

#### Functional disability assessment

Eight instruments were used to assess functional disability. The Oswestry Disability Index (ODI) was the most frequently employed (32 studies, 76%), assessing ten dimensions including pain intensity, mobility, and social engagement (27, 28). Its robust psychometric properties have established it as the gold standard for low back pain-related disability (29). The Roland-Morris Disability Questionnaire (RMDQ, *n* = 7) and Neck Disability Index (NDI, *n* = 2) were applied less frequently, despite their relevance for cervical or upper spine disorders. Other tools, such as the Japanese Orthopaedic Association (JOA) score and General Function Score (GFS), appeared sporadically and were often used without clear justification regarding validity or target population.

#### Quality-of-life assessment

Three instruments were identified for QoL assessment. The SF-36 (*n* = 27) was the most frequently used, offering a broad evaluation of eight domains encompassing physical, emotional, and social functioning. Its comprehensive scope and cross-cultural validation make it well-suited for chronic disease studies (30, 31). In contrast, the EQ-5D (*n* = 10), with five dimensions and a utility score, was commonly employed in cost-effectiveness analyses. Disease-specific instruments such as the QUALEFFO-41 were rarely used (*n* = 1), reflecting limited adoption despite their potential for condition-targeted QoL evaluation.

#### Patient experience assessment

Patient experience was assessed in 16 studies via Likert-type scales evaluating treatment satisfaction and in 10 studies through patient-reported improvement measures. Despite the importance of capturing subjective perceptions, standardized instruments such as the Patient Global Impression of Change (PGIC) and Patient Global Assessment were reported in only one study each. Moreover, details regarding timing of assessment and instrument validation were frequently omitted, limiting the interpretability and comparability of findings.

#### Psychological and mental health assessment

Psychological health was addressed in only six studies, with the HSCL-25 being the most common (*n* = 4). This instrument evaluates depression and anxiety symptoms and has demonstrated strong internal consistency (Cronbach’s *α* > 0.90). Other tools, such as the ZDI, BDI, and FABQ, were used infrequently (1–3 studies each), despite evidence linking psychological distress to prolonged recovery and reduced treatment adherence. Personality-related tools (e.g. Karolinska Scales of Personality and Somatic Perception Questionnaire) were each used once. Overall, the psychological dimension remains significantly underrepresented, despite well-established evidence linking mental health to treatment adherence, pain persistence, and functional recovery.

### Quality of PRO reporting

As summarized in Table 2, overall adherence to PRO reporting guidelines was moderate. In absolute terms, adherence exceeded 50% for trials that designated PROs as primary endpoints, indicating a moderate level of reporting completeness (mean: 64.69%, range: 65.52–75.86%). In contrast, two studies with PROs as secondary or exploratory endpoints showed adherence rates of 50 and 94.44%, respectively. Most trials (*n* = 41, 97.6%) mentioned PROs in the abstract; however, only one study (2.5%) included PROs in the title, explicitly identifying the use of patient-reported outcomes in an RCT context. Reporting of PRO assessment timing was relatively complete, with 97.6% of studies documenting the intended data collection schedule.

**Table 2:** Level of PROs reporting by type of endpoint. Data are presented as *n* (%).

ISOQOL checklist items	PRO endpoint				Total	
	Primary		Secondary or exploratory			
	Yes	No	Yes	No	Yes	No
Title and abstract						
PRO identified as an outcome in the abstract	40 (100.00)	0 (0)	1 (50.00)	1 (50.00)	41 (97.62)	1 (2.38)
Title is explicit to the RCT including a PRO	1 (2.50)	39 (97.50)	/	/	1 (2.50)	39 (97.50)
Introduction, background, and objectives						
PRO hypothesis stated and domain specified	3 (7.50)	37 (92.50)	0 (0)	2 (100.00)	3 (7.14)	39 (92.86)
Introduction includes summary of PRO research	4 (10.00)	36 (90.00)	/	/	4 (10.00)	36 (90.00)
Includes additional details of hypothesis^‡^	1 (2.50)	39 (97.50)	/	/	1 (2.50)	39 (97.50)
Methods						
Mode of administering PRO tool and data collection method	4 (10.00)	36 (90.00)	0 (0)	2 (100.00)	4 (9.52)	38 (90.48)
Rationale provided for choosing PRO instrument	3 (7.50)	37 (92.5)	0 (0)	2 (100.00)	3 (7.14)	39 (92.86)
Evidence of PRO instrument validity and reliability provided or cited	37 (92.50)	3 (7.50)	1 (50.00)	1 (50.00)	38 (90.48)	4 (9.52)
Intended PRO data collection schedule provided	40 (100.00)	0 (0)	1 (50.00)	1 (50.00)	41 (97.62)	1 (2.38)
PROs identified in the trial protocol; *post hoc* analyses identified	12 (30.00)	28 (70.00)	0 (0)	2 (100.00)	12 (28.57)	30 (71.43)
Status of PRO as either a primary or secondary outcome stated*	38 (95.00)	2 (5.00)	0 (0)	2 (100.00)	38 (90.48)	4 (9.52)
Citation provided for original development of PRO instrument	37 (92.50)	3 (7.50)	/	/	37 (92.50)	3 (7.50)
Windows for valid PRO responses specified and justified appropriately for clinical context	39 (97.50)	1 (2.50)	/	/	39 (97.50)	1 (2.50)
Give a power/sample size calculation relevant to PRO based on clinical rationale (e.g. anticipated effect size)	20 (50.00)	20 (50.00)	/	/	20 (50.00)	20 (50.00)
Give evidence of appropriate statistical analysis and statistical significance tests for each PRO hypothesis	40 (100.00)	0 (0)	2 (100.00)	0 (0)	42 (100.00)	0 (0)
Statistical approaches for dealing with missing data explicitly stated	19 (47.50)	21 (52.50)	0 (0)	2 (100.00)	19 (45.24)	23 (54.76)
Extent of missing data stated	39 (97.50)	1 (2.50)	1 (50.00)	1 (50.00)	40 (95.24)	2 (4.76)
State how multiple comparisons have been made	2 (5.00)	38 (95.00)	/	/	2 (5.00)	38 (95.00)
Results						
Flow diagram or description of participants and those lost to follow-up specifically provided for PROs	39 (97.50)	1 (2.50)	1 (50.00)	1 (50.00)	40 (95.24)	2 (4.76)
Reasons for missing data explained	36 (90.00)	4 (10.00)	1 (50.00)	1 (50.00)	37 (88.10)	5 (11.90)
Description of patients’ characteristics including baseline PRO scores provided	40 (100.00)	0 (0)	2 (100.00)	0 (0)	42 (100.00)	0 (0)
Analysis of PRO data accounts for survival differences between treatment groups	4 (10.00)	36 (90.00)	/	/	4 (10.00)	36 (90.00)
Results reported all PRO domains (if multidimensional) and items identified by reference instrument^§^	40 (100.00)	0 (0)	/	/	40 (100.00)	0 (0)
Provided proportion of patients achieving predefined responder definitions	38 (95.00)	2 (5.00)	/	/	38 (95.00)	2 (5.00)
Discussion						
Explicitly discussed limitations of the PRO components	4 (10.00)	36 (90.00)	0 (0)	2 (100.00)	4 (9.52)	38 (90.48)
Discussed generalizability issues uniquely related to PRO results	40 (100.00)	0 (0)	2 (100.00)	0 (0)	42 (100.00)	0 (0)
Discussed clinical significance of PRO findings	40 (100.00)	0 (0)	2 (100.00)	0 (0)	42 (100.00)	0 (0)
Discussed PRO results in the context of other clinical trial outcomes	39 (97.50)	1 (2.50)	2 (100.00)	0 (0)	41 (97.62)	1 (2.38)
Other information						
Include copy of instrument if not previously published (either in the article appendix or in the online version)^†^	38 (95.00)	2 (5.00)	/	/	38 (95.00)	2 (5.00)

* Studies reporting ‘Unclear’ (*n* = 3 in primary PRO endpoint; *n* = 5 in secondary or exploratory PRO endpoint).

^†^ The response options are ‘yes’ and ‘N/A’.

^‡^ Details such as rationale for selected domain(s), expected direction(s) of change, and time points for assessment.

^§^ Including results that are not just statistically significant.

A major methodological gap concerned the reporting of data collection modalities. Only 9.5% of RCTs (*n* = 4) described whether PRO data were collected via paper, telephone, electronic systems, or clinician administration, with just two studies utilizing electronic methods. Lack of clarity in data capture methods raises concerns about data standardization and potential bias. Furthermore, only 7.1% of studies provided a rationale for the selection of specific PRO instruments, despite the availability of disease-specific and validated tools. This absence limits the interpretability and relevance of the findings across patient populations and clinical contexts.

Although 95.2% of studies documented the extent of missing PRO data and 88.1% provided reasons for missingness (e.g. lost to follow-up, withdrawal), only 45.2% of RCTs described the statistical methods used to handle missing data. This lack of methodological transparency is concerning, as inappropriate handling of missing data may lead to biased estimates and compromised statistical power. In addition, most studies failed to account for survival differences or dropout-related biases in their analysis of PRO endpoints – an especially important consideration in trials involving high-risk spinal procedures.

Most studies (*n* = 40, 95.2%) included a participant flow diagram detailing PRO assessment adherence across follow-up time points. All trials addressed the clinical relevance or generalizability of their PRO findings, though this was often done qualitatively rather than through effect size estimates or minimally important difference thresholds. Only four studies (9.5%) explicitly discussed the limitations of PRO assessments in the context of trial design or measurement bias. Based on ISOQOL composite scores, 12 studies (27.2%) met the predefined threshold for high-quality PRO reporting.

### Factors associated with higher quality of PRO reporting

Multivariable linear regression identified no statistically significant predictors of PRO reporting quality (all *P* > 0.05, Table 3). However, several covariates demonstrated consistent directional patterns. Trials in which the primary PRO showed a statistically significant difference between treatment groups exhibited the strongest positive association with reporting quality (*β* = 0.255), followed by studies with multinational designs (*β* = 0.133).

**Table 3:** Multivariable model for the overall quality of PRO reporting.

Variables	*β*	95% CI	*P* value
Year of publication (continued)	0.118	−5.47, 9.68	0.573
Impact factor (continued)	−0.060	−0.12, 0.10	0.793
Multinational study (yes vs no)	0.133	−8.77, 16.72	0.528
Corporate/industry supported (fully or in part) (yes vs no)	−0.184	−11.56, 4.70	0.395
Sample sizes >200 (yes vs no)	0.042	−6.95, 8.42	0.846
PRO endpoint (primary vs secondary/exploratory)	0.124	−12.44, 22.68	0.556
Difference between treatment arms at primary endpoint (yes vs no)	0.255	−2.55, 12.47	0.187
Reported PROs in a subsequent publication (yes vs no)	0.068	−7.56, 10.38	0.750
Type of disease (ref. lumbar degenerative diseases)			
Cervical spine disorders vs lumbar degenerative diseases	0.062	−11.00, 15.21	0.745
Chronic low back pain vs lumbar degenerative diseases	−0.036	−9.82, 8.12	0.848
Other spine disorders vs lumbar degenerative diseases	−0.206	−22.09, 8.08	0.350
Vertebral fractures vs lumbar degenerative diseases	−0.147	−13.98, 7.91	0.575

In contrast, industry sponsorship showed a modest negative association with reporting quality (*β* = −0.184), while reporting PROs in subsequent publications (*β* = 0.068) and journal impact factor (*β* = −0.060) demonstrated only minimal and nonsignificant associations. Among disease categories, trials focusing on cervical spine disorders displayed a slight positive association (*β* = 0.062). These trends, though not statistically significant, highlight potential areas for improvement in PRO methodology, especially in under-resourced or industry-led trials.

## Discussion

This review systematically evaluated the utilization and reporting quality of PROs in RCTs related to spinal disorders. Despite growing recognition of the importance of PROs, our findings revealed marked heterogeneity in instrument selection and persistent gaps in reporting transparency, justification, and methodological rigor – highlighting a disconnect between the ideals of patient-centered research and its practical execution.

The vast majority of studies (95.2%) designated PROs as primary endpoints, indicating growing recognition of their importance in spinal health research. Pain and functional disability instruments – particularly the VAS and ODI – dominated the landscape, reflecting their ease of use and established validity. In contrast, psychological health tools appeared in only six studies, suggesting an underappreciation of the psychosocial dimensions that critically influence recovery and long-term outcomes.

In terms of reporting completeness and transparency, all included studies disclosed baseline PRO scores and subdomain results from multidimensional instruments, thereby mitigating selective reporting bias and enhancing external validity. Notably, even nonsignificant domains (e.g. emotional functioning) were accompanied by full effect estimates and confidence intervals, in alignment with recommendations to reduce over-reliance on *P*-values and promote interpretability (32).

Nonetheless, notable methodological shortcomings persist. Over 90% of trials failed to pre-specify PRO-related hypotheses or define the intended outcome domains (e.g. ‘pain interference’ vs ‘physical function’), raising concerns about post hoc reinterpretation and selective inference. This omission undermines hypothesis-driven research and heightens the risk of data-driven significance claims. Furthermore, the majority of studies did not justify the selection of PRO instruments, thereby undermining reproducibility and the potential for cross-study synthesis.

Statistically, most studies did not implement multiplicity correction when analyzing longitudinal or multidimensional PRO data – contrary to best practices outlined in ICH E9 (33). This omission raises the risk of inflated type I error rates and spurious conclusions.

In addition, few studies incorporated clinically meaningful thresholds such as the minimal clinically important difference (MCID) (34) or Patient Global Impression of Change (PGIC) (35). Without these benchmarks, findings – though statistically significant – often lack clinical clarity, impairing their utility in guiding shared decision-making or setting realistic patient expectations.

Although formal adherence to reporting guidelines has improved, with 27.2% of trials meeting high-quality criteria, deeper issues – such as measurement conceptualization and clinical interpretability – remain underdeveloped. In addition, the lack of statistically significant predictors in our regression may reflect limited sample power or inadequate capture of trial complexity through the selected covariates (36).

In addition, it is noteworthy that our study revealed that the majority of spine-related randomized controlled trials designate PROs as primary endpoints, a pattern that contrasts sharply with other disciplines such as oncology, where PROs are more commonly employed as secondary outcomes. This distinction likely reflects the greater emphasis in spine research on patient-centered outcomes, including pain relief and functional improvement. Consequently, in the context of spinal trials, the methodological rigor and transparency of PRO reporting are particularly critical for the interpretability and comparability of findings, further underscoring the necessity of standardizing PRO design and reporting practices.

To improve methodological rigor, future trials should ground PRO selection in conceptual frameworks and align instrument choice with clinical goals, disease context, and patient priorities. Integration of clinically meaningful thresholds (e.g. MCID, PGIC), transparent handling of missing data, and early engagement of psychometricians and patient partners will enhance both validity and applicability. Expanding sample diversity and conducting subgroup analyses will further support the external generalizability of PRO findings in spinal care.

In brief, while the incorporation of PROs into spinal RCTs has become more routine, major opportunities remain to elevate their methodological quality and clinical relevance. By moving beyond traditional framework adherence and toward conceptually driven, patient-centered implementation, future research can better fulfill the promise of PROs in advancing personalized, value-based spine care.

### Limitations

Despite the study’s rigorous design and inclusion criteria, several limitations should be noted. First, to enable robust multivariate analysis, only RCTs with ≥50 participants were included, potentially excluding smaller trials with informative findings. Second, although the search strategy covered two major databases, relevant unpublished or non-English studies may have been missed. Third, due to heterogeneity across included RCTs, a formal risk of bias assessment was not performed, limiting our ability to examine the relationship between bias and PRO reporting quality.

## Conclusion

In conclusion, while the use of PROs has become increasingly common in spinal randomized controlled trials, substantial opportunities remain to improve their methodological rigor and reporting transparency. Advancing truly patient-centered research in this field requires more than adherence to formal guidelines – it calls for a cultural shift toward integrative, multidisciplinary collaboration and deliberate, theory-driven study design. With these systematic enhancements, PROs can more fully realize their potential in supporting precision medicine, fostering participatory care, and elevating the overall quality of spinal disorder management.

## Supplementary materials



## ICMJE Statement of Interest

The authors declare that there is no conflict of interest that could be perceived as prejudicing the impartiality of the work reported.

## Funding Statement

This work was supported by grants from the National Natural Science Foundation of China (Grant No. 82405611), the Postdoctoral Fellowship Program of CPSF under Grant Number GZC20251369, and the Postdoctor Research Fund of West China Hospital, Sichuan University (Grant No. 2025HXBH015).

## Author contribution statement

WBH, RL, and WHH analyzed and processed the extracted data and participated in the writing of the manuscript. WBH and JYM analyzed and interpreted all the data collected. LD and ZCL revised the framing of the manuscript and were major contributors in writing the manuscript. All of them read and approved the final manuscript.

## Data availability

The data that support the findings of this study are not openly available due to reasons of sensitivity and are available from the corresponding author upon reasonable request.
