# Supplementary material for: Patient-reported outcomes in randomized controlled trials of spinal disorders: a methodological quality assessment and recommendations for future research
Source: EFORT Open Rev. 2026 Jun 1;11(6):627–36. doi: 10.1530/EOR-2025-0171 (PMC13240631; doi:10.1530/EOR-2025-0171)
Supplement: Supplementary file 1 [file supplementary_materials.pdf]

Appendix A – Record of database searches

Medline/PubMed		
**No.**	**Search formula**	**N**
**#1**	"quality of life"[MeSH Terms] OR "life quality"[Title/Abstract] OR "HRQL"[Title/Abstract] OR "QOL"[Title/Abstract] OR "HRQOL"[Title/Abstract] OR "health status"[Title/Abstract] OR "health outcomes"[Title/Abstract] OR "patient outcomes"[Title/Abstract] OR "patient reported symptom"[Title/Abstract] OR "patient reported outcome"[Title/Abstract] OR "PRO"[Title/Abstract] OR "depression"[Title/Abstract] OR "pain"[Title/Abstract] OR "fatigue"[Title/Abstract] OR "anxiety"[Title/Abstract] OR "emotional"[Title/Abstract] OR "psychosocial"[Title/Abstract] OR "psychological"[Title/Abstract] OR "distress"[Title/Abstract] OR "functioning"[Title/Abstract] OR "functional status"[Title/Abstract] OR "social wellbeing"[Title/Abstract] OR "symptom burden"[Title/Abstract] OR "symptom assessment"[Title/Abstract] OR "Sexual"[Title/Abstract]	**2,871,872**
**#2**	"central cord syndrome"[MeSH Terms] OR "spine"[MeSH Terms] OR "spinal cord injuries"[MeSH Terms] OR "spinal cord compression"[MeSH Terms] OR "autonomic dysreflexia"[MeSH Terms] OR "spina*"[Title/Abstract] OR "spine*"[Title/Abstract]	**574,053**
**#3**	"randomized controlled trials as topic"[MeSH Terms] OR "randomized controlled trial"[Publication Type] OR "random allocation"[MeSH Terms] OR "RCT"[Title/Abstract] OR "randomized"[Title/Abstract] OR "randomised"[Title/Abstract]	**1,239,503**
**#4**	"surgical procedures, operative"[MeSH Terms] OR "general surgery"[MeSH Terms] OR "surgical procedures, operative"[MeSH Terms] OR "methods"[MeSH Terms] OR "surger*"[Title/Abstract] OR "operati*"[Title/Abstract] OR "Procedure"[Title/Abstract]	**6,124,500**
**#5**	#1 AND #2 AND #3 AND #4 Filters: from 2005 - 2025	**6471**
**Web of Science**		
**#1**	(((AB=(quality of life OR life quality OR health-related quality of life OR health related quality of life OR HRQL OR QOL OR HRQOL OR health status OR health outcomes OR patient outcomes OR patient reported symptom OR patient reported outcome OR PRO OR depression OR pain OR fatigue OR anxiety OR emotional OR psychosocial OR psychological OR distress OR functioning OR functional status OR social wellbeing OR symptom distress OR symptom burden OR symptom assessment)) AND AB=(central cord syndrome OR spine OR spinal cord injuries OR spinal cord compression OR autonomic dysreflexia OR spina* OR spine*)) AND AB=(randomized controlled trial OR random allocation OR RCT OR randomized OR randomised)) AND AB=(general surgery	**9041**

	OR surgical procedures OR methods OR surger* OR operati* OR Procedure)<br>https://www.webofscience.com/wos/alldb/summary/f3c8777e-1db9-4c7d-8615-10cade4e8939-013c013ec7/relevance/1	

Appendix B. ISOQOL-recommended PRO reporting standards for randomized clinical trials

From: Brundage, M., Blazeby, J., Revicki, D. et al. Patient-reported outcomes in randomized clinical trials: development of ISOQOL reporting standards. Qual Life Res 22, 1161–1175 (2013). https://doi.org/10.1007/s11136-012-0252-1

Reporting standard category	Standards recommended for all studies with a PRO (regardless of whether the PRO is a 1° or 2° outcome)	Additional standards recommended for studies in which the PRO is a 1° outcome
**Title and abstract**		
		The title of the paper should be explicit as to the RCT including a PRO.
	The PRO should be identified as an outcome in the abstract.	
**Introduction, background, and objectives**		
		The introduction should contain a summary of PRO research that is relevant to the RCT.
	The PRO hypothesis should be stated and specify the relevant PRO domain(s) if applicable.	
		Additional details regarding the hypothesis should be provided, including the rationale for the selected domain(s), the expected direction(s) of change, and the time points for assessment.
**Methods**		
	The mode of administration of the PRO tool and the methods of collecting data (e.g., telephone, other) should be described.	
		A citation for the original development of the PRO instrument should be provided.
	The rationale for choice of the PRO instrument used should be provided.	
		Windows for valid PRO responses should be specified and justified as being appropriate for the clinical context.
	Evidence of PRO instrument validity and reliability should be provided or cited.	

Reporting standard category	Standards recommended for all studies with a PRO (regardless of whether the PRO is a 1° or 2° outcome)	Additional standards recommended for studies in which the PRO is a 1° outcome
	The intended PRO data collection schedule should be provided.	
	PROs should be identified in the trial protocol; post-hoc analyses should be identified.	
	The status of PRO as either a primary or secondary outcome should be stated.	
		There should be a power/sample size calculation relevant to the PRO based on a clinical rationale (e.g., anticipated effect size).
	There should be evidence of appropriate statistical analysis and tests of statistical significance for each PRO hypothesis tested.	
		The manner in which multiple comparisons have been addressed should be provided.
	The extent of missing data should be stated†.	
	Statistical approaches for dealing with missing data should be explicitly stated†.	
**Results**		
	A flow diagram or a description of the allocation of participants and those lost to follow-up should be provided for PROs specifically.	
	The reasons for missing data should be explained.	
	The study patients' characteristics should be described, including baseline PRO scores.	
		The analysis of PRO data should account for survival differences between treatment groups if relevant.

Reporting standard category	Standards recommended for all studies with a PRO (regardless of whether the PRO is a 1° or 2° outcome)	Additional standards recommended for studies in which the PRO is a 1° outcome
		Results should be reported for all PRO domains (if multi-dimensional) and items identified by the reference instrument (i.e., not just those that are statistically
		The proportion of patients achieving predefined responder definitions should be provided where relevant.
**Discussion**		
	The limitations of the PRO components of the trial should be explicitly discussed.	
	Generalizability issues uniquely related to the PRO results should be discussed, if applicable.	
	The clinical significance of the PRO findings should be discussed.	
	The PRO results should be discussed in the context of the other clinical trial outcomes.	
**Other information**		
		A copy of the instrument should be included if it has not been published previously

†These items were originally combined in the ISOQOL recommended standards but have been split for the purpose of our work.

Appendix C. List of references

Full list of the 42 articles evaluated in this review.

1. Deer, T. R. et al. The MOTION Study: A Randomized Controlled Trial with Objective Real-World Outcomes for Lumbar Spinal Stenosis Patients Treated with the mild® Procedure: One-Year Results. Pain Med 23, 625–634 (2022).
2. Lurie, J. D. et al. Surgical Versus Nonoperative Treatment for Lumbar Disc Herniation. Spine 39, 3 – 16 (2014).
3. Rodrigues, L. C. L. & Natour, J. Surgical treatment for lumbar spinal stenosis: a single-blinded randomized controlled trial. Adv Rheumatol 61, 25 (2021).
4. Weinstein, J. N. et al. Surgical Versus Nonoperative Treatment for Lumbar Spinal Stenosis Four-Year Results of the Spine Patient Outcomes Research Trial. Spine 35, 1329 – 1338 (2010).
5. Farrokhi, M. R., Alibai, E. & Maghami, Z. Randomized controlled trial of percutaneous vertebroplasty versus optimal medical management for the relief of pain and disability in acute osteoporotic vertebral compression fractures. SPI 14, 561 – 569 (2011).
6. Wardlaw, D. et al. Efficacy and safety of balloon kyphoplasty compared with non-surgical care for vertebral compression fracture (FREE): a randomised controlled trial. The Lancet 373, 1016 – 1024 (2009).
7. Weinstein, J. N. et al. Surgical versus Nonsurgical Therapy for Lumbar Spinal Stenosis. N Engl J Med 358, 794 – 810 (2008).
8. Abdu, W. A. et al. Long-Term Results of Surgery Compared With Nonoperative Treatment for Lumbar Degenerative Spondylolisthesis in the Spine Patient Outcomes Research Trial (SPORT). Spine (Phila Pa 1976) 43, 1619 – 1630 (2018).
9. Benyamin, R. M., Staats, P. S. & MiDAS Encore, I. MILD® Is an Effective Treatment for Lumbar Spinal Stenosis with Neurogenic Claudication: MiDAS ENCORE Randomized Controlled Trial. Pain Physician 19, 229 – 242 (2016).
10. Staats, P. S., Benyamin, R. M., & MiDAS ENCORE Investigators. MiDAS ENCORE: Randomized Controlled Clinical Trial Report of 6-Month Results. Pain Physician 19, 25 – 38 (2016).
11. Malmivaara, A. et al. Surgical or Nonoperative Treatment for Lumbar Spinal Stenosis? Spine 32, 1 – 8 (2007).
12. Whang, P. et al. Sacroiliac Joint Fusion Using Triangular Titanium Implants vs. Non-Surgical Management: Six-Month Outcomes from a Prospective Randomized Controlled Trial. Int J Spine Surg 9, 6 (2015).

13. Klazen, C. A. et al. Vertebroplasty versus conservative treatment in acute osteoporotic vertebral compression fractures (Vertos II): an open-label randomised trial. The Lancet 376, 1085 – 1092 (2010).
14. Chen, D., An, Z.-Q., Song, S., Tang, J.-F. & Qin, H. Percutaneous vertebroplasty compared with conservative treatment in patients with chronic painful osteoporotic spinal fractures. Journal of Clinical Neuroscience 21, 473 – 477 (2014).
15. Yang, Z. et al. Treatment of MM-associated spinal fracture with percutaneous vertebroplasty (PVP) and chemotherapy. Eur Spine J 21, 912 – 919 (2012).
16. Ould-Slimane, M. et al. A prospective multicenter randomized study comparing the SpineJack system and nonsurgical management with a brace in acute traumatic vertebral fractures: the SPICO study. J Neurosurg Spine 40, 790 – 800 (2024).
17. Berenson, J. et al. Balloon kyphoplasty versus non-surgical fracture management for treatment of painful vertebral body compression fractures in patients with cancer: a multicentre, randomised controlled trial. The Lancet Oncology 12, 225 – 235 (2011).
18. Mannion, A. F., Brox, J. I. & Fairbank, J. C. T. Comparison of spinal fusion and nonoperative treatment in patients with chronic low back pain: long-term follow-up of three randomized controlled trials. The Spine Journal 13, 1438 – 1448 (2013).
19. Fairbank, J. et al. Randomised controlled trial to compare surgical stabilisation of the lumbar spine with an intensive rehabilitation programme for patients with chronic low back pain: the MRC spine stabilisation trial. BMJ 330, 1233 (2005).
20. Cesaroni, A. & Nardi, P. V. Plasma disc decompression for contained cervical disc herniation: a randomized, controlled trial. Eur Spine J 19, 477 – 486 (2010).
21. Peul, W. C. et al. Surgery versus Prolonged Conservative Treatment for Sciatica. N Engl J Med 356, 2245 – 2256 (2007).
22. Furunes, H. et al. Total disc replacement versus multidisciplinary rehabilitation in patients with chronic low back pain and degenerative discs: 8-year follow-up of a randomized controlled multicenter trial. The Spine Journal 17, 1480 – 1488 (2017).
23. Lurie, J. D. et al. Surgical Versus Nonoperative Treatment for Lumbar Disc Herniation. Spine 39, 3 – 16 (2014).
24. Kallmes, D. F. et al. A Randomized Trial of Vertebroplasty for Osteoporotic Spinal Fractures. N Engl J Med 361, 569 – 579 (2009).
25. Brox, J. I. et al. Lumbar instrumented fusion compared with cognitive intervention and exercises in patients with chronic back pain after previous surgery for disc herniation: A prospective randomized controlled study. Pain 122, 145 – 155 (2006).
26. Hsu, K. Y. et al. Quality of life of lumbar stenosis – treated patients in whom the X STOP interspinous device was implanted. SPI 5, 500 – 507 (2006).
27. Engquist, M. et al. A 5- to 8-year randomized study on the treatment of cervical

radiculopathy: anterior cervical decompression and fusion plus physiotherapy versus physiotherapy alone. SPI 26, 19–27 (2017).
28. Weinstein, J. N. et al. Surgical vs Nonoperative Treatment for Lumbar Disk Herniation. JAMA 296, 2441 (2006).
29. Lee, S., Zheng, H., Park, S.-M., Kim, H.-J. & Yeom, J. S. A Randomized Controlled Trial of Vertebral Body Decompression Procedure Versus Conservative Treatment for Painful Vertebral Compression Fracture. Medicina 59, 1848 (2023).
30. Kadaňka, Z. et al. Conservative treatment versus surgery in spondylotic cervical myelopathy: a prospective randomised study. European Spine Journal 9, 538–544 (2000).
31. Ivar Brox, J. et al. Randomized Clinical Trial of Lumbar Instrumented Fusion and Cognitive Intervention and Exercises in Patients with Chronic Low Back Pain and Disc Degeneration. Spine 28, 1913–1921 (2003).
32. Fritzell, P., Hägg, O., Wessberg, P. & Nordwall, A. 2001 Volvo Award Winner in Clinical Studies: Lumbar Fusion Versus Nonsurgical Treatment for Chronic Low Back Pain. Spine 26, 2521–2532 (2001).
33. Weinstein, J. N. et al. Surgical Compared with Nonoperative Treatment for Lumbar Degenerative Spondylolisthesis. The Journal of Bone and Joint Surgery-American Volume 91, 1295–1304 (2009).
34. Hellum, C. et al. Surgery with disc prosthesis versus rehabilitation in patients with low back pain and degenerative disc: two year follow-up of randomised study. BMJ 342, d2786–d2786 (2011).
35. Hedlund, R., Johansson, C., Hägg, O., Fritzell, P. & Tullberg, T. The long-term outcome of lumbar fusion in the Swedish lumbar spine study. The Spine Journal 16, 579–587 (2016).
36. Slätis, P. et al. Long-term results of surgery for lumbar spinal stenosis: a randomised controlled trial. Eur Spine J 20, 1174–1181 (2011).
37. Anderson, P. A., Tribus, C. B. & Kitchel, S. H. Treatment of neurogenic claudication by interspinous decompression: application of the X STOP device in patients with lumbar degenerative spondylolisthesis. SPI 4, 463–471 (2006).
38. Masoudi, M. S., Haghnegahdar, A., Ghaffarpasand, F. & Ilami, G. Functional Recovery Following Early Kyphoplasty Versus Conservative Management in Stable Thoracuolumbar Fractures in Parachute Jumpers. Clin Spine Surg 30, E1066–E1073 (2017).
39. Desai, M. J. et al. A Prospective, Randomized, Multicenter, Open-label Clinical Trial Comparing Intradiscal Biacuplasty to Conventional Medical Management for Discogenic Lumbar Back Pain. Spine (Phila Pa 1976) 41, 1065–1074 (2016).

40. Weinstein, J. N. et al. Surgical Versus Nonoperative Treatment for Lumbar Disc Herniation. Spine 33, 2789 – 2800 (2008).
41. Weinstein, J. N. et al. Surgical versus Nonsurgical Treatment for Lumbar Degenerative Spondylolisthesis. N Engl J Med 356, 2257 – 2270 (2007).
42. Delitto, A. et al. Surgery Versus Nonsurgical Treatment of Lumbar Spinal Stenosis. Ann Intern Med 162, 465 – 473 (2015).

Appendix D. Detailed characteristics of each RCT

t author-year	Main Country	Type of disease-new	Overall study sample size	Multinational study	Difference between treatment arms in the primary endpoint	Reported PROs in a subsequent publication	Indu supp
tto 2015	USA	Lumbar Degenerative Diseases	169	No	No	No	No
nstein 2007	USA	Lumbar Degenerative Diseases	301	No	No	Yes	No
nstein 2008	USA	Lumbar Degenerative Diseases	473	No	Yes	Yes	No
i 2016	USA	Other Spine Disorders	63	No	Yes	No	Yes
oudi 2017	Iran	Vertebral Fractures	70	No	Yes	No	No
erson 2006	USA	Lumbar Degenerative Diseases	75	No	Yes	No	No
s 2011	Finland	Lumbar Degenerative Diseases	94	No	Yes	No	No
und 2016	Sweden	Chronic Low Back Pain	294	No	Yes	No	No
ts 2016	USA	Lumbar Degenerative Diseases	302	No	Yes	Yes	No
um 2011	Norway	Chronic Low Back Pain	172	No	Yes	No	No
nstein 2009	USA	Lumbar Degenerative Diseases	304	No	Yes	Yes	Yes
ell 2001	Sweden	Chronic Low Back Pain	294	No	Yes	No	Yes
k 2003	Norway	Chronic Low Back Pain	64	No	No	Yes	No
anka 2000	Czech Republic	Cervical Spine Disorders	48	No	No	No	No
2023	Korea	Vertebral Fractures	98	No	No	No	Yes
nstein 2006	USA	Lumbar Degenerative Diseases	472	No	No	Yes	No
vamin 2016	USA	Lumbar Degenerative Diseases	302	No	Yes	Yes	No
quist 2017	Sweden	Cervical Spine Disorders	59	No	Yes	No	No
2006	USA	Lumbar Degenerative Diseases	191	No	Yes	No	Yes
k 2006	Norway	Lumbar Degenerative Diseases	60	No	No	Yes	No
mes 2009	USA	Vertebral Fractures	131	Yes	No	No	Yes
e 2014	USA	Lumbar Degenerative Diseases	474	No	No	No	No
nes 2017	Norway	Chronic Low Back Pain	172	No	Yes	No	No
2007	Netherlands	Other Spine Disorders	283	No	No	No	No
roni 2010	Italy	Cervical Spine Disorders	115	No	Yes	No	Yes
bank 2005	UK	Chronic Low Back Pain	349	No	Yes	Yes	Yes
nion 2013	UK	Chronic Low Back Pain	473	Yes	No	No	No
nson 2011	USA	Vertebral Fractures	129	Yes	Yes	No	Yes
l-Slimane 2024	France	Vertebral Fractures	95	No	Yes	No	Yes
g 2012	China	Vertebral Fractures	76	No	Yes	No	No

t author-year	Main Country	Type of disease-new	Overall study sample size	Multinational study	Difference between treatment arms in the primary endpoint	Reported PROs in a subsequent publication	Indu supp
n 2014	China	Vertebral Fractures	89	No	Yes	No	No
en 2010	Netherlands	Vertebral Fractures	202	No	Yes	No	Yes
ng 2015	USA	Other Spine Disorders	148	No	Yes	No	No
mivaara 2007	Finland	Lumbar Degenerative Diseases	94	No	Yes	No	No
u 2018	USA	Lumbar Degenerative Diseases	304	No	Yes	No	No
nstein 2008	USA	Lumbar Degenerative Diseases	278	No	Yes	Yes	No
dlaw 2009	UK	Vertebral Fractures	300	Yes	Yes	No	Yes
okhi 2011	Iran	Vertebral Fractures	82	No	Yes	No	Yes
nstein 2010	USA	Lumbar Degenerative Diseases	278	No	Yes	No	No
riguesLCL 2021	Brazil	Lumbar Degenerative Diseases	63	No	No	No	No
e 2014	USA	Lumbar Degenerative Diseases	474	No	Yes	No	No
2022	USA	Lumbar Degenerative Diseases	155	No	Yes	No	Yes

Appendix E. Categorization and frequency of included PRO measurement tools

Name of the Instruments	Scope of Application	Psychometric Properties / Validation Summary	Fre
**Assessment:**			
Analog Scale for Pain (VAS)	A scale that measures pain intensity from no pain to worst pain imaginable.	Widely validated	
: Pain Rating Scale (NPRS)	A numerical scale (0-10) for patients to rate their pain intensity.	Strong validity, reliability, and responsiveness	
w Back Pain Bothersomeness Scale)	Assesses how bothersome lower back pain symptoms are.	Well-validated	
atica Bothersomeness Index (SBI)	Measures the severity and impact of sciatica symptoms.	Acceptable validity; moderate evidence in sciatica cohorts.	
nosis Bothersomeness Index (StBI)	Evaluates the discomfort level caused by spinal stenosis.	Limited but supportive validation data in lumbar stenosis studies.	
Pain Bothersomeness Scale (LPBS)	Assesses how much leg pain interferes with daily life.	Limited psychometric evidence; used mainly in trial settings.	
**tional Disability Assessment:**			
y Low Back Pain Disability Index	Measures the degree of disability caused by lower back pain.	Gold-standard instrument with strong reliability, validity, and responsiveness in lumbar spine populations.	
Morris Disability Questionnaire)	Assesses disability levels in patients with lower back pain.	Well-validated	
neral Function Score (GFS)	Evaluates the overall functional ability of a patient.	Demonstrates acceptable reliability; used in spine research despite limited condition-specific validation.	
Back Disability Question	A general measure of back-related disability.	Simple global measure; moderate evidence for construct validity in musculoskeletal conditions.	
e Orthopaedic Association score	Assesses neurological function in spinal disorders.	Widely used; good reliability and responsiveness in cervical and lumbar myelopathy populations.	
ky performance status (KPS) score	Measures the functional status of patients with serious illnesses.	Widely used; good reliability and responsiveness in cervical and lumbar myelopathy populations.	
sability Index (NDI)	Evaluates how neck pain affects daily activities.	Well-validated with good reliability, construct validity, and responsiveness in neck pain research.	
udy of Osteoporotic Fractures - es of Daily Living (SOF-ADL)	Assesses daily living activities in osteoporotic patients.	Acceptable validity for functional assessment; limited evidence in spinal disorder populations.	

Name of the Instruments	Scope of Application	Psychometric Properties / Validation Summary	Fr
ty of Life Assessment:			
Short Form Survey (SF-36)	General health survey evaluating physical and mental health.	Extensive validation; strong reliability and construct validity across musculoskeletal and spinal populations.	
n Quality of Life-5 Dimensions)	Measures general health status and quality of life.	Widely validated; good utility and responsiveness, especially for economic evaluations.	
ality of Life Questionnaire of the n Foundation for Osteoporosis EFFO)	Measures quality of life in osteoporosis patients.	Well-validated for vertebral fracture-related QoL; limited use in general spinal disorders.	
nt-Reported Outcomes Assessment:			
ion with Treatment (Likert Scale)	Measures patient satisfaction with their treatment.	Face-valid and widely accepted	
Self-Reported Improvement (Likert	Assesses a patient's perceived improvement after treatment.	Simple global rating; moderate validity for capturing subjective treatment benefit.	
Global Assessment (Likert scale)	A subjective measure of overall health status.	Established global item with adequate construct validity in chronic conditions.	
Global Impression of Change (PGIC)	Evaluates perceived change in health status over time.	Widely used; good face validity and sensitivity to patient-perceived improvement.	
Perceived Recovery (Likert Scale)	Assesses a patient's perception of their recovery.	Good discriminative ability; limited formal validation but commonly used in musculoskeletal trials.	
nological and Mental Health Assessment:			
symptom check list (HSCL-25)	Screens for anxiety and depression symptoms.	Strong reliability and validity across cultures; widely used in clinical and research settings.	
's Fear-Avoidance Belief nnaire (FABQ)	Assesses fear-related avoidance behaviors in chronic pain patients.	Well-validated with good psychometric performance in chronic low back pain populations.	
g Depression Scale (ZDI)	A self-rating scale for depression severity.	Adequate reliability and construct validity	
epression Inventory (BDI)	A commonly used tool for measuring depression symptoms.	Gold-standard with excellent reliability, validity, and sensitivity to change.	
ska Scales of Personality (KSP)	Evaluates personality traits related to mental health.	Acceptable psychometric basis; limited application in spinal disorder research.	
Perception Questionnaire (SPQ)	Assesses awareness of bodily sensations and somatic symptoms.	Good internal consistency; used primarily in psychosomatic and pain-related research.	
